# Contact Printing of Multilayered Thin Films with Shape Memory Polymers

**DOI:** 10.1021/acsnano.1c11607

**Published:** 2022-03-30

**Authors:** Soyoun Kim, Nan Liu, Alexander A. Shestopalov

**Affiliations:** Department of Chemical Engineering, University of Rochester, Rochester, New York 14625, United States

**Keywords:** transfer printing, shape memory polymer, thin-film printing, adhesion modulation, donor substrate

## Abstract

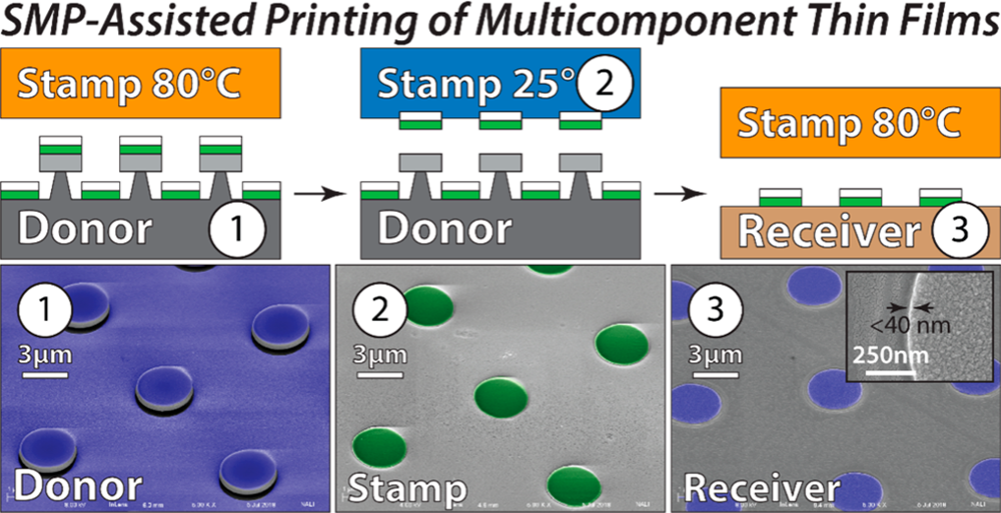

This study describes a method for transfer printing microarrays of multilayered organic–inorganic thin films using shape memory printing stamps and microstructured donor substrates. By applying the films on the microstructured donor substrates during physical vapor deposition and modulating the interfacial adhesion using a shape memory elastomer during printing, this method achieves (1) high lateral and feature-edge resolution and (2) high transfer efficiency from the donor to the receiver substrate. For demonstration, polyurethane-acrylate stamps and silicon/silicon oxide donor substrates were used in the large-area transfer printing of organic–inorganic thin-film stacks with micrometer lateral dimensions and sub-200 nm thickness.

## Introduction

In recent years, contact transfer printing has been considered a promising manufacturing technique for the assembly and patterning of micro-/nanoscale objects. It uses an elastomeric stamp to pick up an ink material from a donor substrate and then deposit it onto a receiver surface. In another iteration, the functional inks are directly deposited onto the stamp without the use of the donor substrate. Contact printing is intrinsically compatible with both top-down and bottom-up device manufacturing. As the traditional top-down approaches face compatibility challenges with atomically precise bottom-up processes (*e.g.*, atomic layer deposition and etching), contact printing is gaining a renewed interested in semiconductor device manufacturing as a technique that can generate self-aligned patterns for future bottom-up electronic devices.^1^ In addition, contact transfer printing is often suggested as a complementary technique to traditional photolithography because it is compatible with a wider set of materials (organic, inorganic, biological, self-assembled monolayers, colloidal, polymeric, *etc*.) and because of its ability to pattern flexible and nonplanar surfaces in a scalable and cost-efficient manner.^2−6^ Another attractive feature of the transfer printing is a potential to replicate patterns with high lateral and vertical resolution. The resolution in contact printing is primarily limited by the ink diffusion and the deformations of the elastomeric stamp material. By selecting solid inks with a low vapor pressure and by relying on stiff elastomers, contact printing can replicate sub-micrometer features and features with sub-100 nm edge resolution at a fraction of the cost of deep UV photolithography.^7,8^

Examples of applying transfer printing in microelectronics and photonics include fabrication of light-emitting displays,^7,9−12^ photovoltaic cells,^13,14^ and photoconductors^15,16^ where functional materials are prefabricated on donor interfaces and vertically integrated onto receiver substrates. More recently, contact printing has been considered in semiconductor device fabrication for the assembly of multilayered, fully integrated devices. This process enables single-step, large-area patterning of multicomponent structures with reduced alignment requirements and no diffraction/diffusion limits associated with the photolithography and shadow mask deposition. Contact printing is also compatible with flexible and nonplanar substrates in addition to organic semiconductors and inorganic light-emitting materials that cannot be structured using traditional photolithography.^17−21^ However, the examples of transfer printing of heterogeneous multilayer microfeatures are rare and often limited to sequential layer-by-layer thin-film assembly due to the problems associated with the integration of dissimilar materials.^22−25^

**Figure 1:**
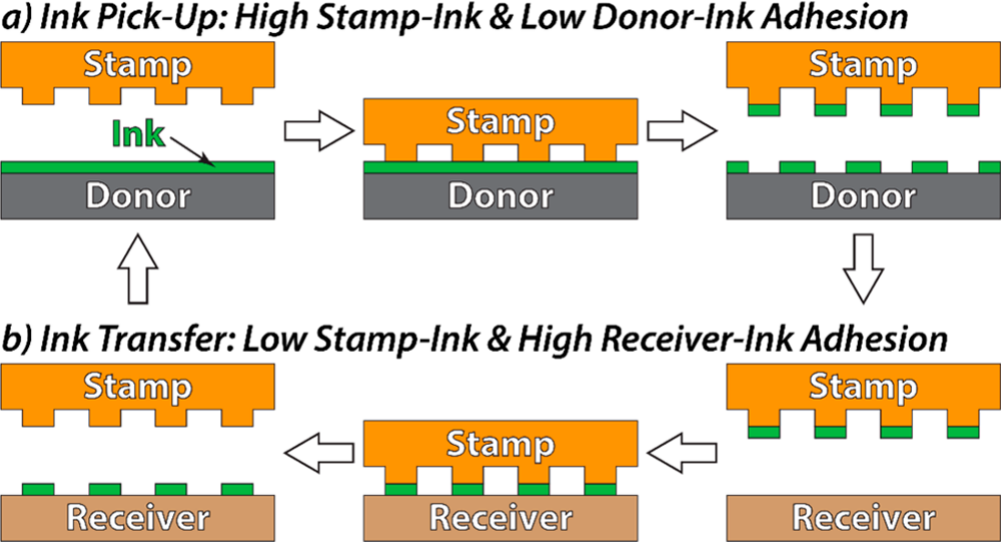
(a, b) Contact transfer printing of an ink material (green) from a donor substrate (gray) to a receiver surface (brown) using an elastomeric stamp (orange).

As the association and disassociation of inks with stamps and donor/receiver substrates rely primarily on noncovalent interactions, the key factor for a successful and complete printing process of heterogeneous materials is a precise control over interfacial adhesion at the stamp-ink and ink-donor/receiver interfaces during the pick-up and transfer. The adhesion between the ink and the stamp needs to be greater than the adhesion between the donor and the ink during the pick-up, while it should be less than the adhesion between the ink and the receiver during the transfer step (Figure 1). One way to achieve this requirement is to chemically modify the stamp polymer to adjust its surface energy for a particular donor/ink/receiver combination. For example, several studies describe modifications of stamp materials such as polydimethylsiloxane (PDMS), polyolefin plastomers (POPs), Kraton elastomeric, and polyurethane acrylates (PUAs) to render them compatible with various hydrophilic and hydrophobic inks.^8,26,27^ However, most of these examples are limited to half-printing cycles (*e.g*., where the ink is directly deposited on the stamp and subsequently transferred onto the receiver without the pick-up step). They also require optimization of the polymer composition to achieve the desired level of surface hydrophobicity. It is also possible to adjust the surface energy of the stamp without changing its composition by using release/sacrificial layers.^28−33^ However, this approach limits the variety of the inks to materials that are immiscible with the sacrificial layers and can also result in contamination of the printed interface with the releasing layer molecules.

Researchers have also explored various approaches to achieve a stimuli-responsive interfacial adhesion to modulate the strength of noncovalent interactions during the ink pick-up and transfer. A common and effective approach to such switchable adhesion is to vary the stamp contact kinetics that influences the degree of interfacial adhesion in viscoelastic materials. For example, the Rogers group and others have demonstrated that adhesion in transfer printing can be modulated by applying directional load and varied rates of peel to promote adhesion switching, owing to the viscoelastic nature of the PDMS stamps.^34−38^ Alternatively, external triggers such as electromagnetic switching induced by magnetic fields or electrostatic forces can be used to actuate micro-/macrostructured surfaces to change their areas of contact. For example, Linghu *et al*. created a stamp with reservoirs filled with magnetic particles, which push and bulge the thin surface membrane to promote delamination,^39^ while Kim *et al.*’s method utilizes dense fibrils of dielectric-coated carbon nanotubes, which increase adhesion by 100-fold by applying 30 V with their compliance and the long-range electrostatic attraction.^40^ Furthermore, thermally induced phase transitions are often exploited to control changes in adhesive forces via a change in contact quality and mechanics. Eisenhaure *et al.* have performed various examples of dry adhesive assembly of inks composed of Si, SiO_2_, Au, and SU8 using microstructured shape memory polymers (SMPs), achieving high adhesion by freezing the temporarily increased contact area created with reduced Young’s modulus while assisting detachment with the release of the stored elastic energy during the shape recovery phase.^41−43^ While most of these methods have successfully demonstrated the macroassembly of ink layers with feature dimensions in the hundreds of micrometers, they face challenges in transferring small sub-10 μm features such as (1) invariability of the interfacial energy release rate from the contact kinetics at small scales, (2) manufacturing problems in making small multicomponent stamp features that incorporate stimuli-responsive materials, or (3) incompatibility with both classes of inorganic (hard) and organic (soft) ink materials.

In this work, we present a method for contact printing of organic–inorganic multilayer features using a microstructured donor substrate and a flat shape memory polymer stamp. The donor substrate serves as a patterning template for microfeatures with high edge resolution. As the target features are evaporated onto the elevated platforms, the thin-film inks do not require additional patterning steps or masks and are separated from the background upon deposition. The thermomechanical shape-memory indentation of the flat stamp against the donor substrate locks in the conformal contact between the stamp and the multilayered features during the pick-up step. During the transfer step, the recovery of the indentations helps release the features onto the receiving substrate. Specifically, we demonstrate a transfer of 140 nm multilayered thin-film stacks in the form of dense 3.7 μm circular features over a 5 × 5 mm^2^ size area. Additionally, we show that the donor substrates and stamps can be regenerated and reused. We also analyze the resolution, material transfer completeness, and the overall transfer efficiency over the total areas of the donor and receiver substrates. We show that the printed features were transferred with sub-50 nm edge resolution and that multicomponent features with layers as thin as 40 nm can be replicated without defects. Our control experiments demonstrate that the thermomechanical programming of the shape memory polymer stamp is a key element in enabling a full cycle of the material transfer and that without it the transfer is ineffective.

## Results and Discussion

### Donor Substrate

The transfer printing of rigid inorganic materials presents a more significant challenge than the printing of soft organic thin films. The stiffness of a continuous inorganic layer prevents clean separation/breakage of the features from the rest of the film during the pick-up step. Continuous inorganic films are either completely delaminated from the donor surface when pulled off by an adhesive stamp or randomly broken into irregularly shaped pieces near the stamp-film contact edges.^44^ Thus, the prepatterning of the inorganic and/or stiff multilayered ink films is necessary and can be done either by shadow mask lithography or by using the structured donor substrates.

Shadow mask lithography patterns the film using physical vapor deposition (PVD) through a microscale hole array. Because the mask is separated by a micrometer gap, the material diffusion in the area between the mask and the substrate limits the resolution of the deposited features to ∼5–10 μm.^45^ Another alternative is to deposit the film onto the array of photolithographically defined features on the donor substrates. However, because the evaporated material deposits onto the walls of the donor structures, the deposited film on the top of the features is not separated from the background, preventing its clean release during the pick-up step.

**Figure 2:**
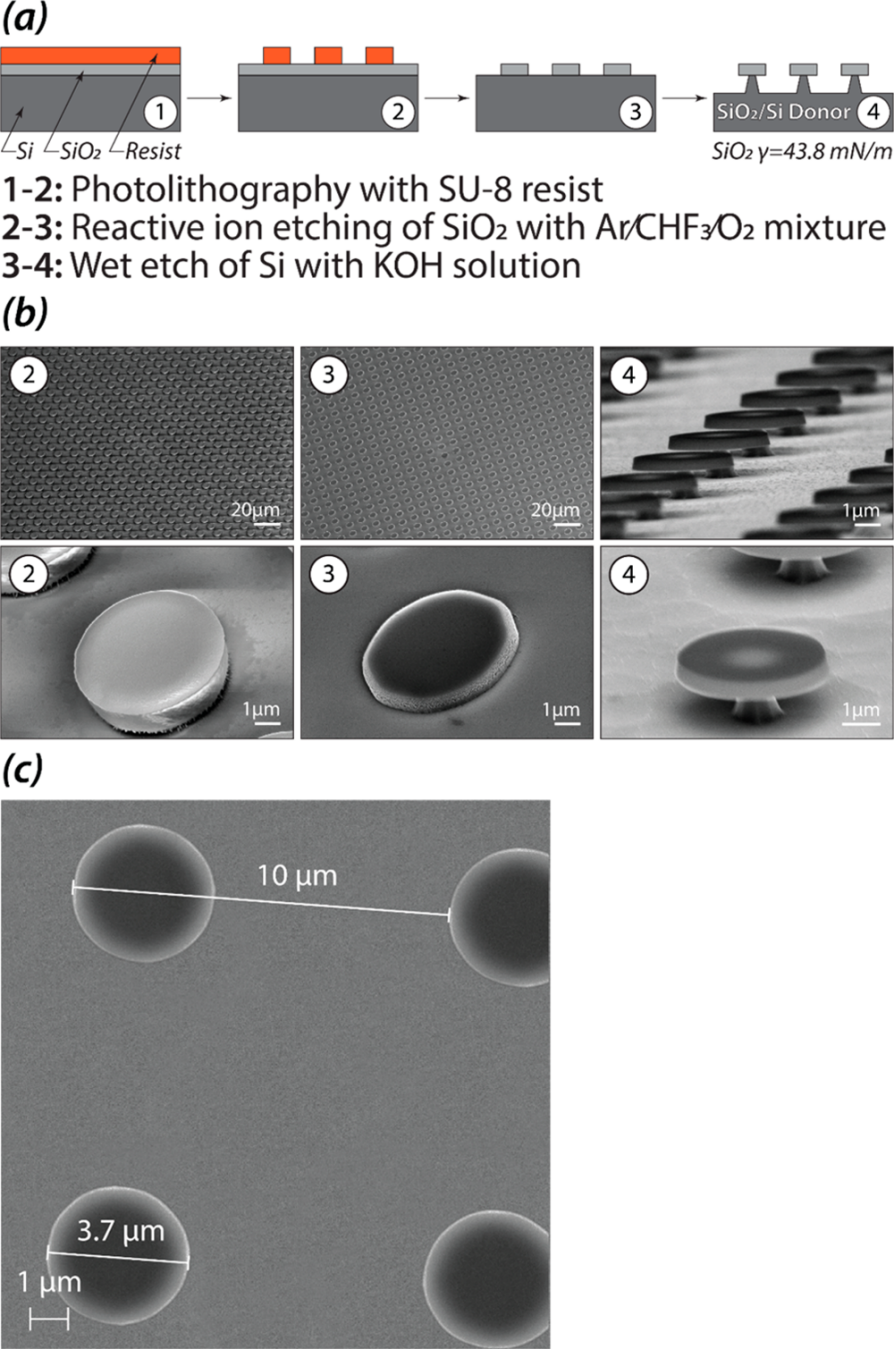
(a) Fabrication of the donor substrate; (b) tilted SEM images of the donor substrate features after photolithography (2), RIE (3), and KOH etch (4); (c) vertical SEM image of the final donor substrate features.

To overcome this limitation, we developed a donor substrate with undercut, mushroom-like structures that enable separation of the deposited ink features from the background during the PVD step. Analogous mushroom-like features were reported in different applications like adhesion enhancement,^46−48^ localized surface plasmon resonance (LSPR) sensing,^49,50^ super-omniphobic surface,^51−53^ and whispering gallery devices.^54,55^ However, many of the reported structures were fabricated using soft organic materials unsuitable for contact printing applications, and so far, such structures have not been considered as structuring masks for prepatterning PVD films. Our fabricated structures contain a silica flat platform to provide the transferring sites for the ink films and a silicon pillar to elevate the platform from the surface (Figure 2). Such geometry (1) ensures separation of the ink features from the background and (2) overcomes the diffusion limitation of the shadow mask deposition by eliminating the gap between the ink feature and the substrate. Because the structures are made from rigid and inert silicon and silica layers, they can withstand vertical compressive and pulling forces during the ink pick-up and can be cleaned and regenerated after a printing cycle.

The donor substrate fabrication is shown in Figure 2a. First, a pattern of SiO_2_ microdisks (3.7 μm diameter, 500 nm height) was prepared on a silicon wafer using photolithography (step 1–2) and RIE (step 2–3). After Nanostrip and oxygen plasma cleaning, concentrated KOH solution was used to selectively remove silicon and make undercut silicon oxide features (step 3–4). The extent of the undercut can be controlled by the KOH etch time and temperature.

**Figure 3:**
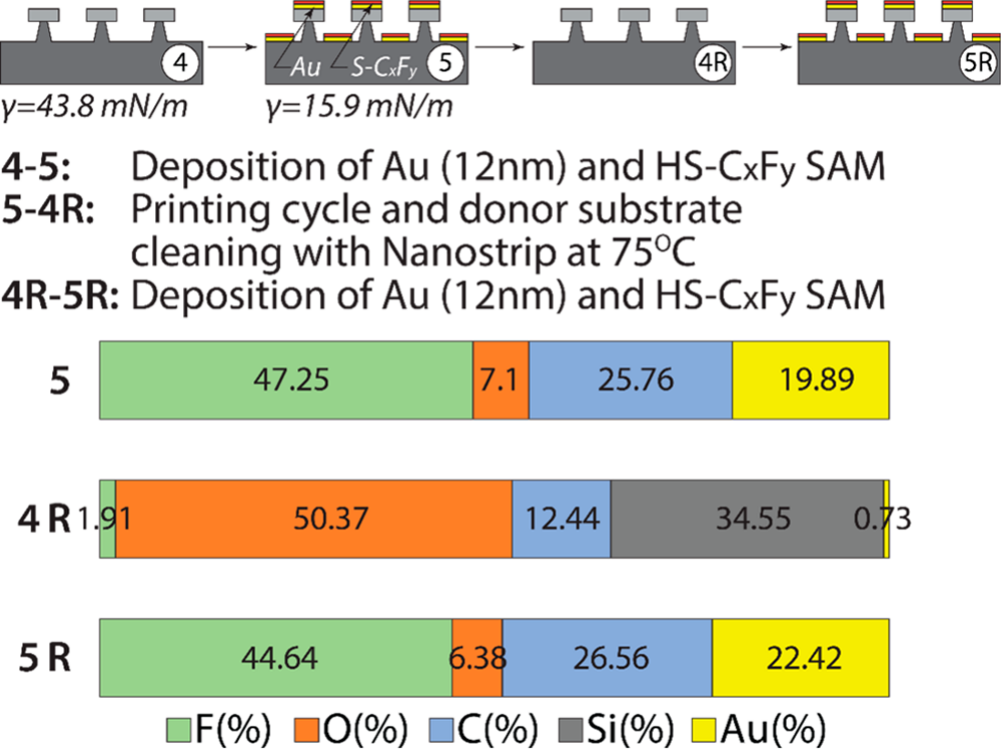
Preparation and regeneration of the donor substrate. XPS analysis of the regenerating cycle: the atomic composition (atomic percent ratio) of the donor interface after functionalization with the SAM on gold (5), after the printing cycle and cleaning (4R), and after the redeposition of the SAM on gold. The substrates 5 and 5R contain F and Au atoms from the SAM on gold. The cleaned substrate (4R) shows only traces of Au and F atoms confirming successful removal of the SAM and gold layers.

Figure 2b,c shows tilted and vertical images of the resulting donor substrate features. The features occupy 10.75% of the donor surface area (3.7 μm diameter discs separated by 10 μm). The surface energy (43.8 mN/m, water contact angle) and roughness (4.5 nm, AFM) of the donor substrate were measured on the corresponding flat samples that had the same composition as the top interface of the donor substrate features. Because of the high surface energy of the silica layer (43.8 mN/m), the donor substrate was sputtered with a thin layer of gold (12 nm) and functionalized with a 1*H*,1*H*,2*H*,2*H*-perfluorodecanethiol solution in isopropanol to create a hydrophobic self-assembled monolayer (SAM) that lowers the surface energy (15.9 mN/m) while maintaining low roughness (5.2 nm, Figure 3, step 4 → 5). Because the SAM is covalently attached to the sputtered gold surface, it does not migrate during transfer printing. The successful surface treatment was verified by XPS with an increase in the F 1s signal and the absence of the Si 2p signal (Figure 3).

We showed that the donor substrate can be regenerated and reused after a complete printing cycle of an inorganic–organic ink film (Figure 3, steps 5-5R). After the printing, the donor substrate was immersed in a Nanostrip solution at 75 °C for 1 h to remove the remaining ink film and the Au-SAM layers, exposing clean silica and silicon interfaces (Figure 3, substrate 4R). Subsequently, the donor substrate was refunctionalized with the Au-SAM layers yielding a material that had the same surface composition as the freshly prepared donor substrate (Figure 3, substrates 5 and 5R). This regenerated donor substrate was reused again in the printing experiments.

### SMP Stamp

**Figure 4:**
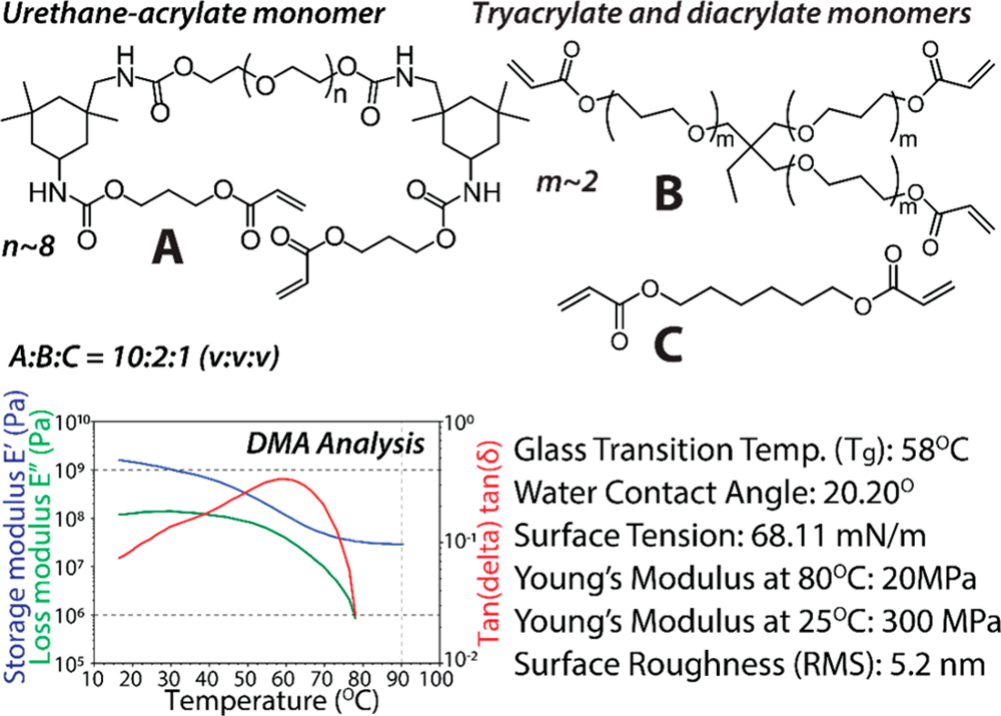
Composition of the prepolymeric mixture: monomers A, B, and C form a cross-linked covalent network after a UV-induced polymerization of the acrylate groups. DMA analysis of the polymerized PUA_G_ material. Physical properties of the PUA_G_ SMP stamp obtained from the DMA analysis, goniometry, and AFM.

Shape memory polymers (SMPs) are a class of materials that can adopt and hold an arbitrarily deformed shape and then recover its initial form through the application of external stimuli such as heat, light, or electromagnetic fields.^56^ Thermal SMPs are by far the most common and studied type of shape memory material. These polymers can effectively hold a temporarily deformed shape by undergoing a temperature-induced phase transition from a rubbery to a glassy or semicrystalline state, resulting in a significant increase in the Young’s modulus of the material. A large stiffness change and an insignificant volume change of glassy SMPs during the phase transition make them promising materials for switchable dry adhesive stamps in micro-/nanoscale printing applications. For this study, we selected an urethane-acrylate polymer (PUA_G_) as our SMP material due to its suitable glass transition temperature (*T*_g_) above room temperature (*T*_g_ ≈ 58 °C), its more than 10-fold increase in elasticity modulus (*E*′_rubbery_ ≈ 20 MPa, *E*′_glassy_ ≈ 300 MPa), the exhibited good moldability with negligible volume change, and the material’s low surface roughness.^8^ The chemical structures of the polymer components are shown in Figure 4.

DMA analysis was performed to measure the glass transition temperature and stiffness of the SMP stamp in its glassy and rubbery states. Upon reaching the glass transition, the Young’s modulus of the PUA_G_ increases by an order of magnitude. AFM analysis and water contact angle measurements were performed to determine surface roughness and surface energy of the molded SMP polymer at 25 °C (Figure 4). Our expectation was that above the glass transition temperature, PUA_G_ would uniformly conform to the ink interface when the holding pressure is applied, but the conformality would fail when the holding force was removed due to its high elasticity and relatively low surface energy. However, when the conformal contact is maintained during the phase transition into a glassy state, the significant increase in stiffness would prevent the stamp recovery into its original undeformed state, stabilizing the van der Waals contact (used in the pick-up step from the donor substrate). During the transfer step, thermal energy would reverse the effect and break the van der Waals interactions between the ink and stamp, enabling the ink release.

**Figure 5:**
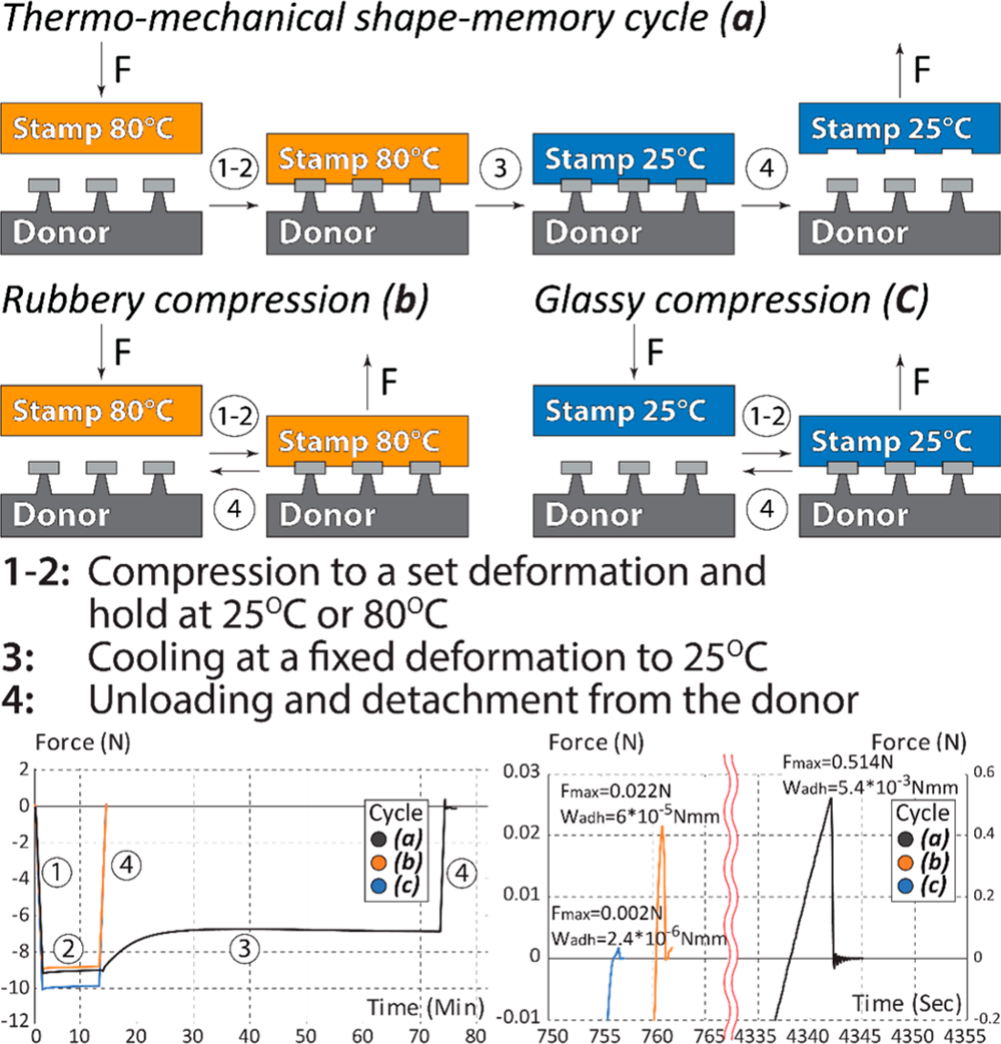
Adhesive interactions between the PUA_G_ stamp and the donor substrate. (a) A shape-memory cycle: the stamp is deformed into the donor substrate at *T* > *T*_g_, cooled below the *T*_g_, and separated from the donor substrate at *T* < *T*_g_; (b, c) control experiments: the stamp is deformed and separated from the donor substrate without undergoing phase transition at *T* > *T*_g_ (b) and at *T* < *T*_g_ (c). The adhesive force and the work of adhesion for all three experiments were calculated from the force–time plots when the stamps were in tension (positive force values).

To demonstrate that the thermomechanical programming of the SMP polymer can be used to modulate its adhesive interactions, we measured the pull-off force and the work required to separate the stamp from the patterned Si/SiO_2_ donor substrate. We first measured the adhesive interactions in the rubbery and glassy states by compressing and detaching the stamp from the donor features at *T* > *T*_g_ and *T* < *T*_g_, respectively (Figure 5b,c). For the thermomechanical shape-memory cycle, we first compressed the elastic stamp into the donor features at *T* > *T*_g_, cooled it to *T* < *T*_g_ under the applied compressive load, and then separated it from the donor substrate (Figure 5a). Both rubbery and glassy compressions achieved small levels of adhesion indicated by the pull-off force required to separate the stamp from the donor and the overall work of adhesion (*F*_MAX_ = 0.022 N, *W*_ADH_ = 2.36 × 10^–6^ N mm at *T* > *T*_g_ and *F*_MAX_ = 0.002 N, *W*_ADH_ = 6 × 10^–5^ N mm at *T* < *T*_g_). At *T* > *T*_g_, the low adhesive contact can be explained by the elastic energy deposited into the stamp during the compression that acts to restore the original geometry of the stamp interface when the compressive load is removed. The low adhesion at *T* < *T*_g_ is likely attributed to the inability of the stamp interface to deform and achieve a high surface area van der Waals contact with the donor substrate due to its increased stiffness.

After a thermomechanical shape-memory cycle, we observed a significant increase in both the pull-off force and the work of adhesion required to separate the polymer from the donor substrate (Figure 5a, *F*_MAX_ = 0.514 N, *W*_ADH_ = 5.4 × 10^–3^ N mm). We attribute this change in adhesion to the geometrical changes on the stamp interface and the formation of conformal contact between the stamp and the donor substrate, which is stabilized by the stiffening glass transition during the cooling of the stamp to *T* < *T*_g_.

**Figure 6:**
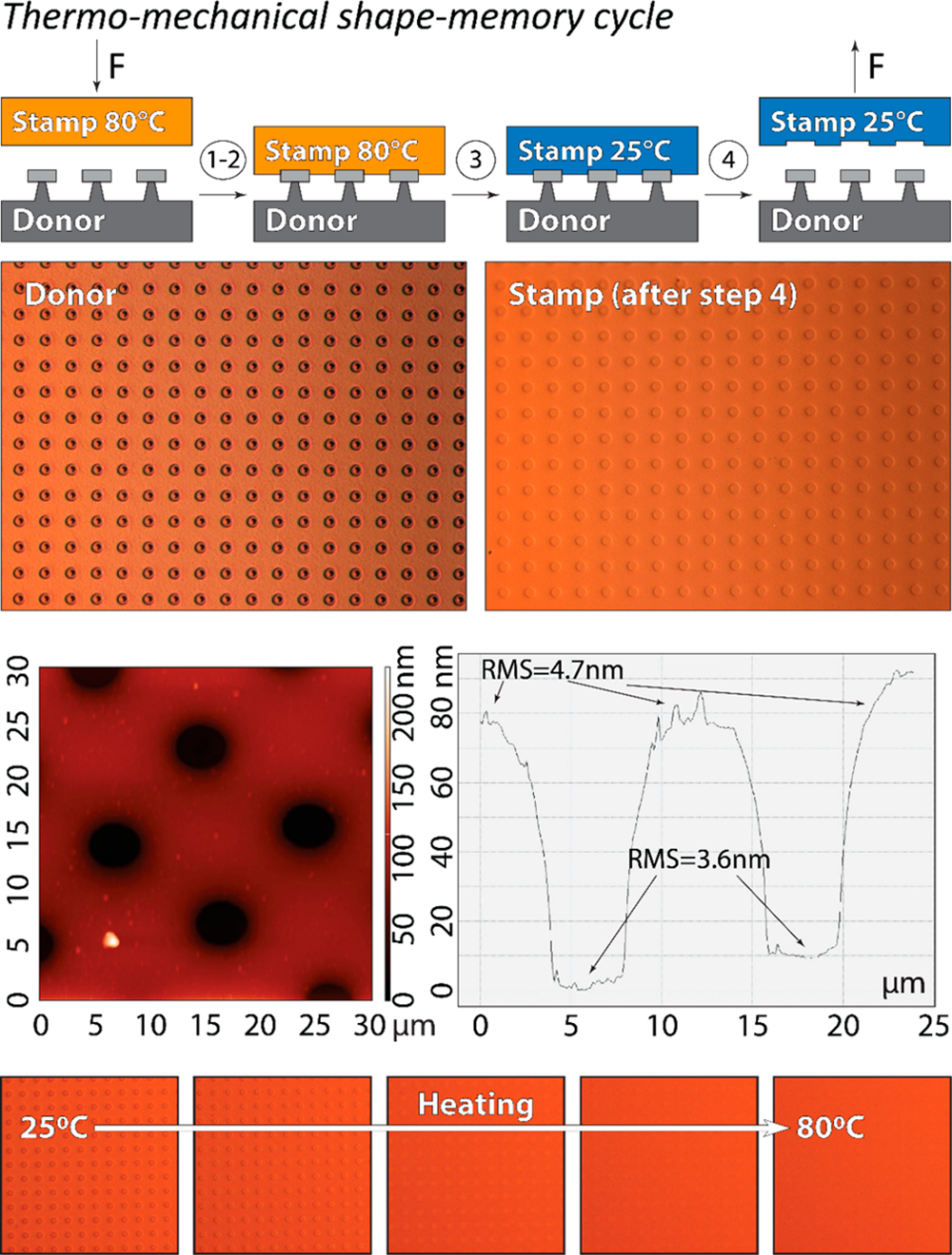
Stabilized features in the PUA_G_ stamp after thermomechanical shape-memory cycle. Optical micrographs of the Si/SiO_2_ donor substrate and the PUA_G_ stamp after it was released from the donor at *T* < *T*_g_. An AFM image and a surface profile of the indented features on the PUA_G_ stamp. Sequential optical micrographs of indentations on the PUA_G_ stamp upon heating. The indentations completely disappear at *T* = 80 °C.

The suggested mechanism of adhesion modulation was supported by the formation of indentations in the stamp interface after the thermomechanical cycle (Figure 6). The dimensions of the stamp features are comparable to that of the circular features on the donor substrate. AFM analysis shows that the imprinted features on the stamp surface exhibit a slightly lower surface roughness than the rest of the stamp interface (Figure 6, AFM line scan).

Upon heating the stamp to *T* > *T*_g_, the imprinted features disappeared, and the stamp returned to its original undeformed shape. These results suggest that the thermomechanical shape-memory programming of the stamp interface can be used to control interfacial adhesion. This change in adhesion is attributed to the formation of a high surface area conformal contact between the stamp and the donor substrate under the applied load at *T* > *T*_g_ and the stabilization of this contact upon cooling to *T* < *T*_g_.

### Transfer Printing Experiments

**Figure 7:**
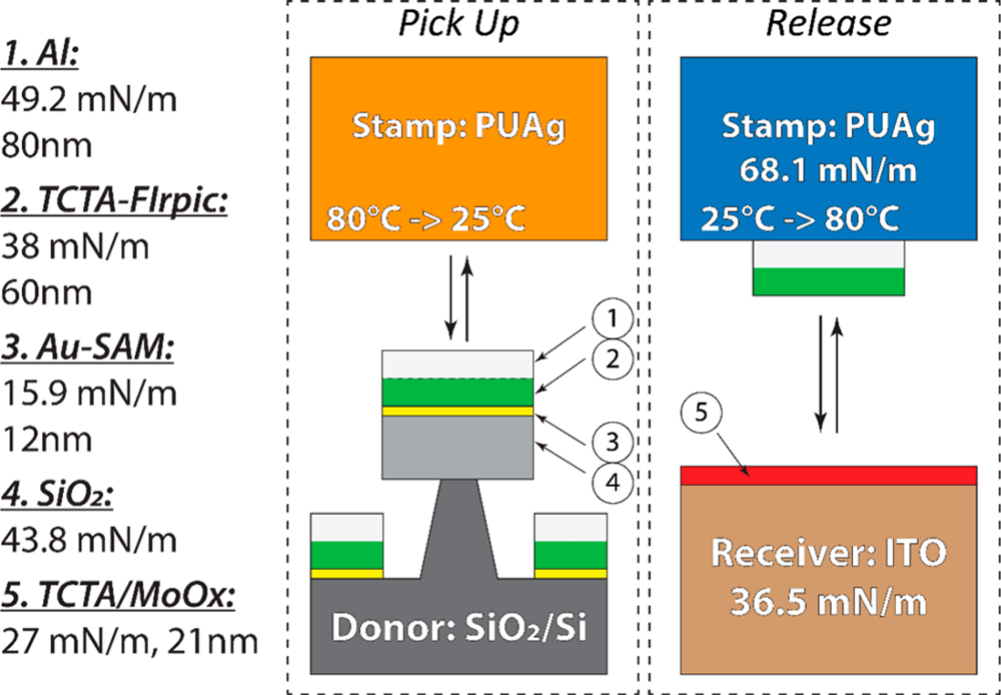
Donor substrate, ink layers, and receiver substrate components and surface energies.

The main goal of this study was to demonstrate selective and accurate large-area transfer of the multilayer thin films from the donor substrate to the receiver substrate using a shape-memory stamp and thermomechanical programming of the adhesive interactions. The overall transfer summary and the thin-film stack components are described in Figure 7.

We used a stack of TCTA doped with FIrpic (60 nm with 30 v/v %) and Al (80 nm) thin films as the printing ink materials. The first layer consisted of TCTA-FIrpic (60 nm, 38 mN/m), codeposited as the organic component. This material is commonly applied as an emitting layer in OLED displays. The fluorescence imaging of this layer can be used to assess the efficiency of the material transfer by monitoring the initial and residual fluorescence before and after TCTA-FIrpic removal. The second layer was made up of an Al (80 nm, 49.2 mN/m) thin film as the inorganic component, which is typically applied as an electrode in various thin-film electronic devices. The initial surface energy of the untreated SiO_2_ donor substrate was 43.8 mN/m, but after treatment with Au-SAM, the surface energy decreased to 15.9 mN/m, assisting in the release of the evaporated films during the pick-up step. The receiver substrate was prepared with MoO_3_ (1 nm), a common hole injection layer, followed by TCTA (20 nm) to match the surface energy of the donor interface with the transferring inks (27 mN/m and 38 mN/m correspondingly).

During the ink pick-up step, conformal contact between the stamp in the glassy state and the aluminum film of the ink will have higher adhesion than a contact between the SAM-functionalized donor surface and the TCTA-FIrpic layer of the ink (68.1 mN/m–49.2 mN/m vs 15.9 mN/m–38 mN/m). Therefore, the layers are expected to separate at the SAM/TCTA–FIrpic interface. During the transfer step, the lowest surface energy contact will be between the TCTA-FIrpic layer of the ink and the TCTA layer of the receiver substrate (38 mN/m–27 mN/m). Thus, to enable the transfer, the conformality of the stamp-ink substrate must be reduced by a phase transition into the rubbery state where the restored elastic energy can be applied to separate the materials.^57^

**Figure 8:**
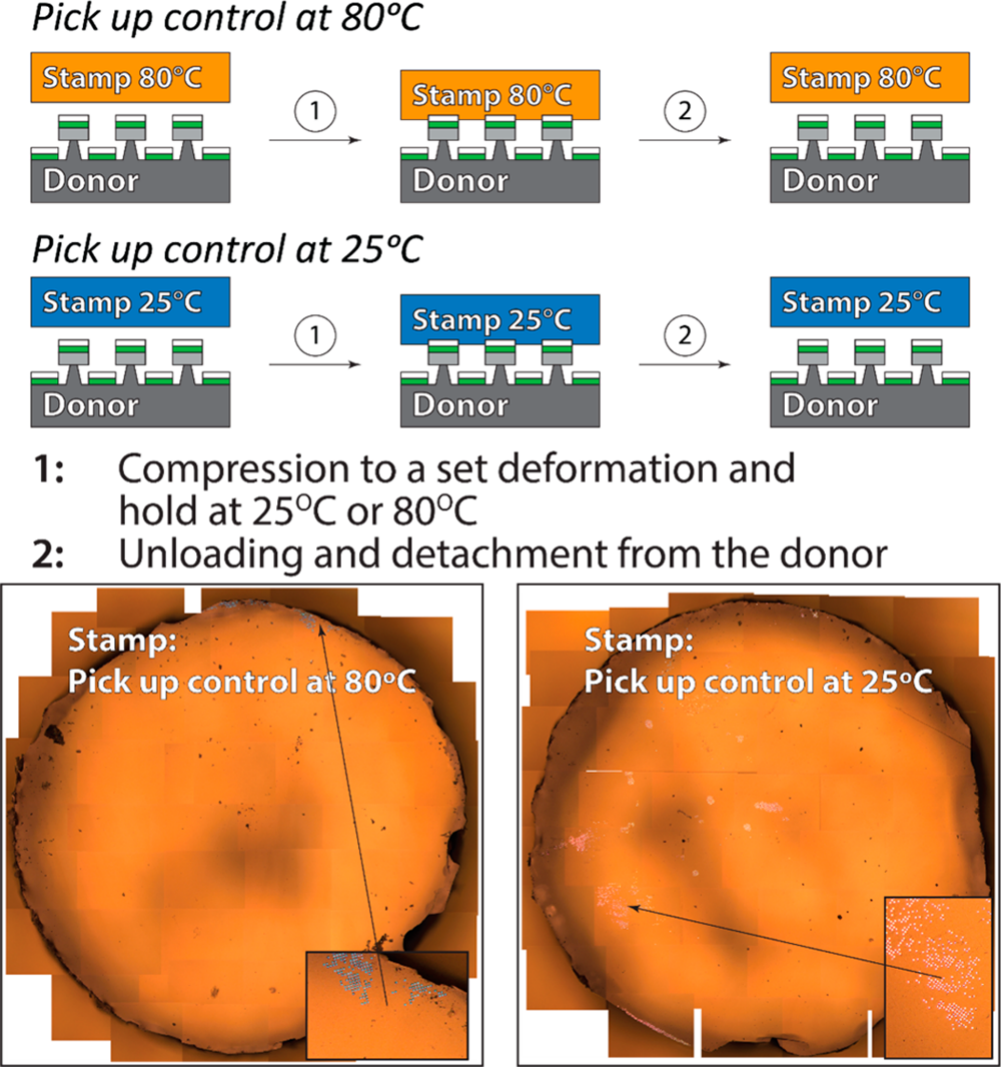
Top: control pick-up experiments with rubbery and glassy flat stamps without the phase transition; bottom: stitched optical micrographs of the stamp surface after the detachment from the donor substrate (∼5 × 5 mm^2^ area). Magnified insets show examples of areas with partially picked-up features (less than 5% of the total contacted area).

We conducted two control experiments at 80 and 25 °C, where the flat PUA_G_ stamps were held in conformal contact with the donor substrates bearing TCTA-FIrpic/Al layers and then detached at the same temperatures (Figure 8). In both cases, less than ∼5% of the features were removed from the donor surface after the stamp detachment. These experiments suggest that the thermomechanical shape-memory programming is necessary to enable successful material transfer and that the stiffening phase transition that occurs under the compressive load during the cooling of the stamp is the key element in this process.

**Figure 9:**
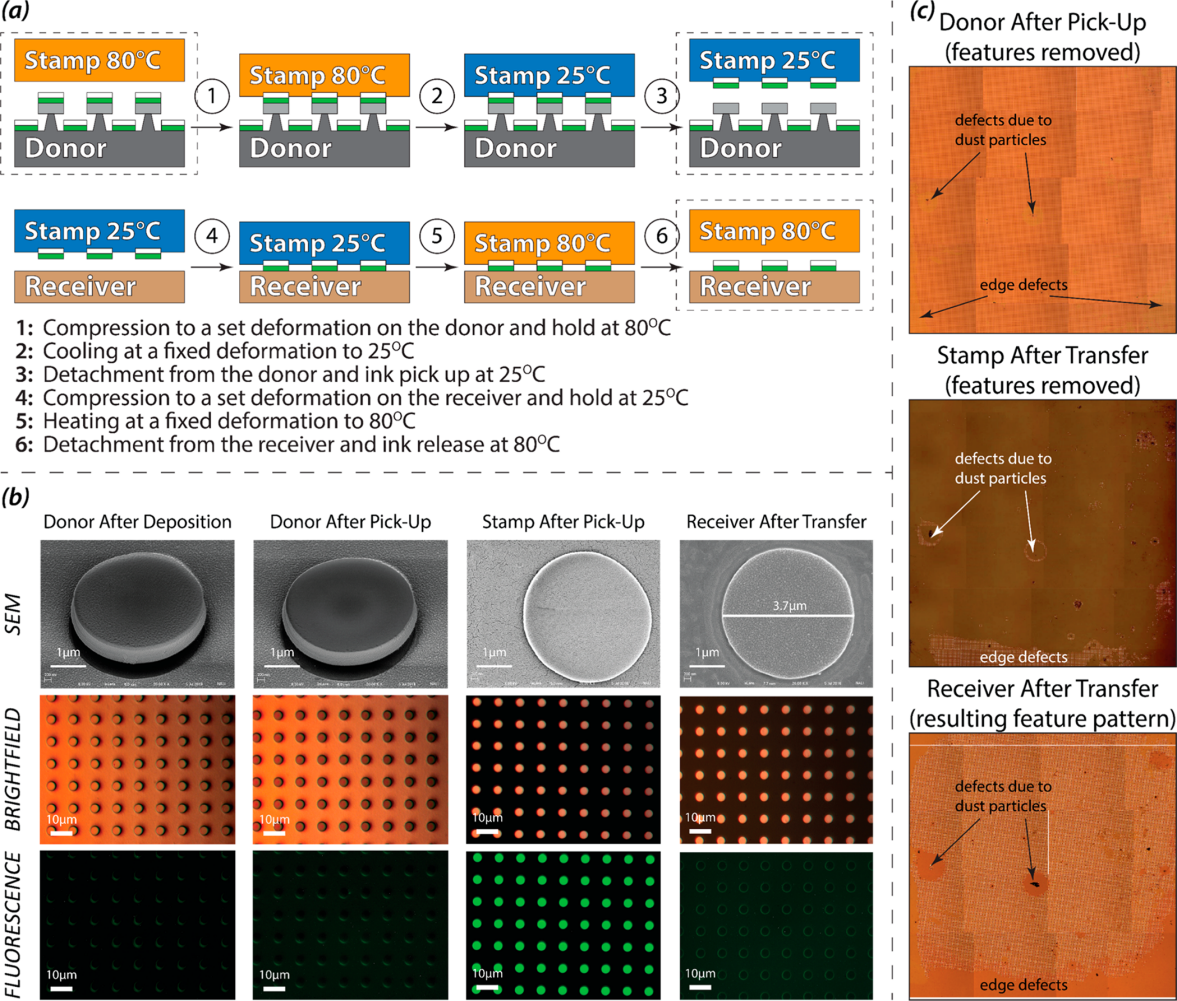
(a) Steps and components of the shape-memory-assisted contact printing cycle; (b) SEM, brightfield (BF) and fluorescent (FL) micrographs of the donor substrate before and after the pick-up step, the stamp after the pick-up step, and the receiver substrate after the transfer step; (c) stitched optical micrographs of the entire donor substrate after the pick-up, the stamp after the transfer, and the receiver substrate after the transfer (∼5 × 5 mm^2^ area).

Figure 9a shows the steps of the complete contact printing protocol. First, the TCTA-FIrpic/Al stack was deposited onto the donor substrate using thermal evaporation. The flat PUA_G_ stamp was then compressed into the donor substrate at 80 °C (Step 1, rubbery state). While the compressive load was maintained, the stamp was cooled to 25 °C (Step 2, glassy state) and separated from the donor, lifting off the TCTA-FIrpic/Al stack (Step 3). Subsequently, the stamp at 25 °C with the attached TCTA-FIrpic/Al features was contacted with the receiver substrate—an ITO electrode covered with a thin layer of TCTA (Step 4). While this contact was maintained, the stamp was heated to 80 °C (Step 5) and then detached from the receiver plate leaving behind a pattern of TCTA-FIrpic/Al features on the receiver surface (Step 6).

**Figure 10:**
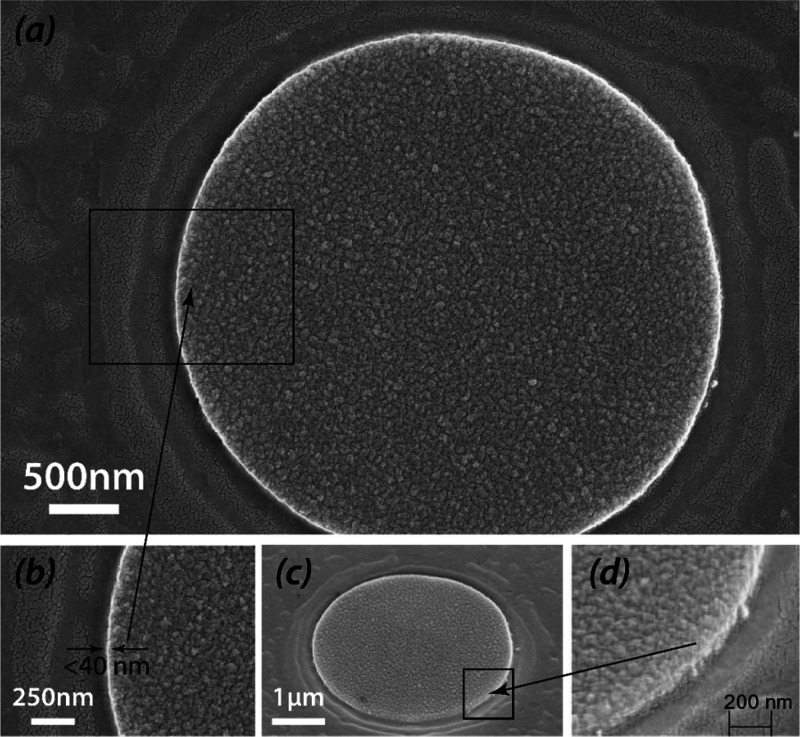
(a) Vertical SEM image of the printed feature on the receiver substrate; (b) magnified SEM image of the printed feature showing the edge resolution; (c) tilted SEM image of the printed feature on the receiver substrate; (d) magnified tilted SEM image of the printed feature showing the height uniformity of the feature material near the edge.

Figure 9b shows SEM images and optical micrographs of the donor substrate before and after the thin-film pick-up, the stamp with picked-up features, and the receiver substrate after the final release step. Brightfield (BF) and fluorescent (FL) microscopy together with SEM imaging were used to confirm successful pattern transfer from the donor to the receiving substrate. The BF mode is suitable for identifying reflective Al films, while the FL micrographs show the presence of the fluorescent TCTA-FIrpic material. Before the pick-up, the features on the donor substrate have the same brightness as the background, while after the pick-up they are darker confirming the aluminum layer removal from the top of the donor features. The FL micrographs of the donor substrate show no fluorescence before or after the pick-up confirming that the TCTA-FIrpic layer under the aluminum layer was completely removed from the donor substrate. The BF images of the stamp after the pick-up clearly show a reflective aluminum layer under a thin and transparent TCTA-FIrpic layer. The FL images of the stamp show fluorescent features confirming the presence of the TCTA-FIrpic layer. The BF features on the receiver substrate after the transfer show high reflectivity confirming the presence of the aluminum layer, while the receiver features on the FL micrographs only show slight fluorescence from the edges because the TCTA-FIrpic layer is covered by the reflective Al layer. Together, the BF and FL images confirm that both TCTA-FIrpic and aluminum layers were completely transferred from the receiver substrate to the donor surface without layer separation or partial material transfer. The tilted SEM images of the donor substrate before and after the printing also show that the TCTA-FIrpic/Al ink layers were removed from the donor features. SEM images of the transferred features on the stamp and the receiver substrate show that the features were not distorted during the printing and that they maintained their dimensions and geometry. The edge resolution of the printed features is below 50 nm (Figure 10a,b). The height of the printed features is uniform near the feature edge confirming that that both layers were transferred completely from the donor to the receiver substrate (Figure 10c,d). These results suggest that the developed technique can be applied to transfer much smaller features and that it is not limited by the material diffusion during the feature/background differentiation on the donor substrate (a common limitation of the deposition through the shadow mask).

To examine the efficiency of the developed technique in a large-area pattern replication, we imaged the entire patterned area (∼5 × 5 mm^2^) of the donor, stamp, and receiver substrates after the printing to construct stitched micrographs (Figure 9c). The efficiency of each printing step was calculated using ImageJ to determine the ratios between the successfully processed area and the no-transfer area. Table 1 shows that ∼68% of the features were successfully transferred from the donor substrate to the stamp and ∼90% of the features from the stamp to the receiver surface. The existence of printing defects is mostly correlated with the presence of dust particles that preclude conformal contact between the stamp and the donor or receiver plates. Another source of printing defects stems from an incomplete attachment of the stamp to the receiver plate at the pattern edge due to lateral substrate misalignment. Such problems can be avoided in more controlled environments (all reported experiments were conducted in a typical wet chemistry lab) and using more precise optomechanical alignment and force distribution systems.

**Table 1:** Pick-up and Transfer Efficiency of Shape Memory-Assisted Contact Printing Calculated with Areas Processed by ImageJ

pick-up area (mm^2^)	transfer area (mm^2^)	total stamp area (mm^2^)
16.1	14.6	23.7

pick-up efficiency	transfer efficiency	total transfer efficiency
68%	90%	61%

**Figure 11:**
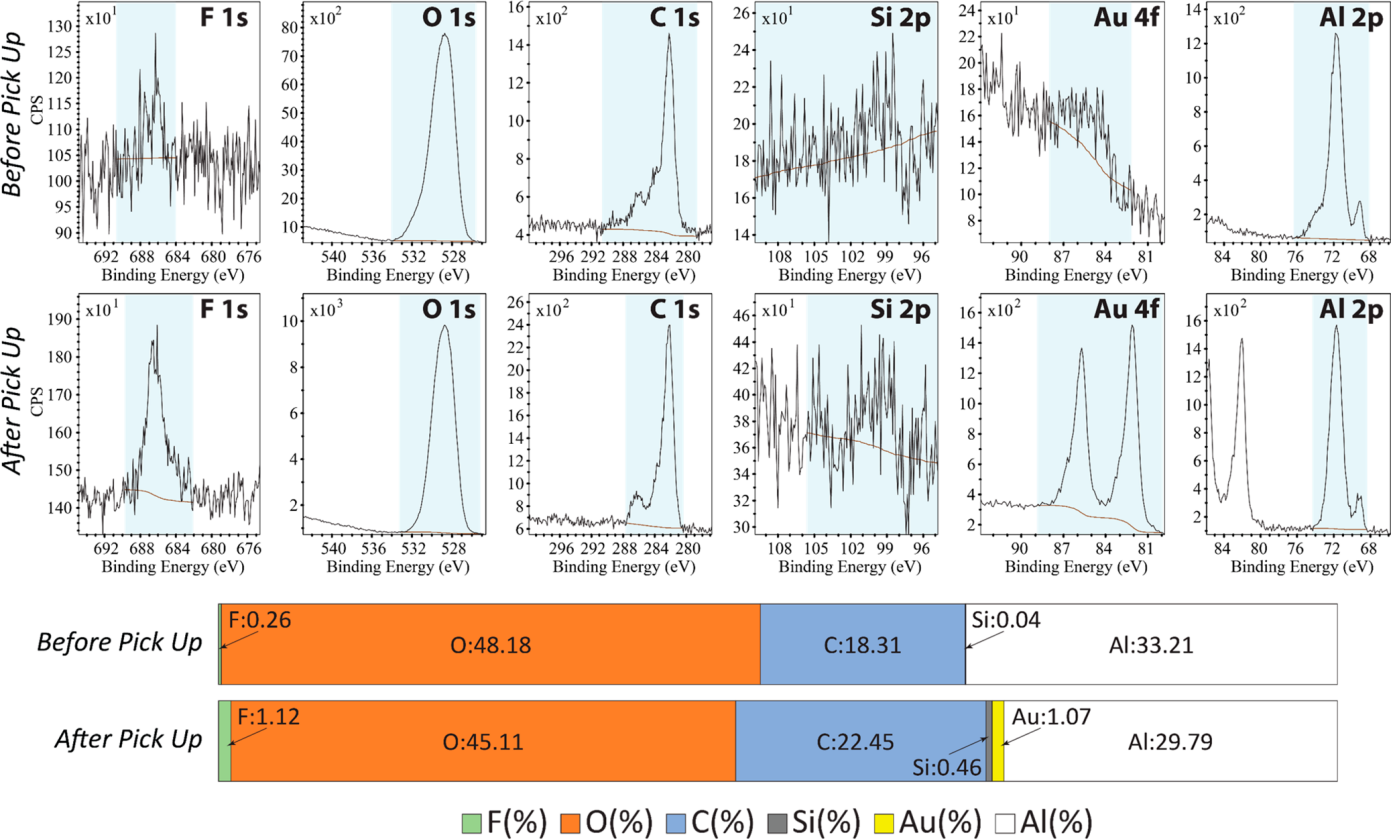
XPS spectra and elemental composition of the donor substrate before and after pick-up (atomic percent ratio).

XPS analysis was conducted to confirm that the whole TCTA-FIrpic/Al stack was completely transferred from the donor substrate to the stamp without delamination or partial material transfer and that the SAM-Au layer on the donor surface was not picked-up by the stamp. Figure 11 shows region scans of F 1s, O 1s, C 1s, Si 2p, Au 4f, and Al 2p atoms on the donor substrate before and after the pick-up step.

Before the pick-up step, the donor surface is composed of oxygen and aluminum atoms in the oxidized aluminum layer and of carbon atoms from randomly physiosorbed organic species. Both F 1s and Au 4f signal intensities are very low because the Au-SAM layer is covered by the TCTA-FIrpic/Al films. After the pick-up, the concentration of Al atoms decreases, while the F 1s, C 1s, and Au 4f signals all increase, confirming the removal of the TCTA-FIrpic/Al layers and the unmasking of the Au-SAM interface that contains dense per-fluorinated organic molecules. The XPS measurements also indicate that the concentration of the Al atoms on the donor surface decreases by ∼10.3% after the pick-up step, which is in good agreement with the pattern density of the transferring sites: π(3.7 μm/2)^2^/100 μm^2^ = 10.75%.

## Conclusions

In this paper, we present the transfer printing of microscale metal–organic thin-film features using shape memory effects and a platform donor substrate. By controlling adhesive interactions between the SMP stamp and the bilayer features, our protocol enables clean and efficient feature transfer without layer delamination or geometrical distortion. Because the platform structure of the donor substrate works as a template during the thin-film fabrication, this technique allows fabrication of high-edge resolution (sub-50 nm) microarrays of the metal–organic thin film. The elevated platform also permits the use of flat SMP stamps, and it aids in controlling adhesive interactions by concentrating the shape memory effect onto the microindented area. These results serve as a promising starting point for future studies targeted to manufacture complete electronic devices by transferring the entire multilayered thin-film devices in one printing cycle and comparing the electrical performance of the printed and vacuum-deposited devices.

## Experimental Section

### Materials

#### Donor Substrate

Negative photoresist SU-8, developer (MicroChem), and Si (100) wafers with a 500 nm thick thermal oxide layer (Universitywafer) were used in photolithography to make microdisk arrays for the donor substrates. Nanostrip (VWR), 7:1 buffer oxide etch (BOE, VWR), KOH solution (Fisher Chemical, 30%, wt %) and 1*H*,1*H*,2*H*,2*H*-perfluorodecanethiol solution (Sigma, 100 mmol/L in isopropanol) were used to manufacture the undercut features of the donor substrate and to modify its surface energy.

#### PUA_G_ Stamp

Poly(ethylene glycol) (PEG, avg *M*_n_ 400), isophorone diisocyanate (IPDI, 98%), tin(II) 2-ethylhexanoate (92.5%), 4,4′-methylenebis(2,6-di-*tert*-butylphenol) (98%), hydroxypropyl acrylate (mixture of isomers, contains 200 ppm inhibitor, 95%), trimethylolpropane propoxylate triacrylate (avg *M*_n_ 644), 1,6-hexanediol diacrylate (HDODA, 80%), 1-hydroxycyclohexyl phenyl ketone (99%), and 2-hydroxy-2-methyl-propiophenone (97%) were acquired from Sigma-Aldrich.

#### Thin-Film Ink

Tris(4-carbazoyl-9-ylphenyl)amine (TCTA, sublimed), bis(3,5-difluoro-2-(pyridin-2-yl)phenyl)(picolinoyloxy)iridium (FIrpic, 99%), aluminum (Al, 99.999%), and molybdenum(VI) oxide (MoO_3_, 99.97%) were obtained from KODAK, Nichem Fine Technology, Kurt J. Lesker, and Sigma-Aldrich, respectively. Indium tin oxide (ITO, 100 nm, 15 Ω/sq)-coated glass substrates were custom ordered from Tinwell Electronics and used as a receiving substrate.

### Equipment

Photolithography mask aligner (OAI 200 Mask Aligner) was used with a Cr photomask (photo science, 5 μm diameter circles array with 5 μm spacing) to fabricate the donor substrate.

Reactive ion etcher (RIE, South Bay Technology Reactive Ion Etcher RIE-2000) was used to etch the SiO_2_ layer of the donor substrate and to clean the substrate with O_2_ plasma.

Dynamic mechanical analysis (TA Instruments RSA G2) was used to measure the glass transition temperature and Young’s modulus of the PUA_G_ material. Temperature sweep data were acquired on a thin-film elastomer (6 mm × 30 mm × 0.25 mm) from 10 to 90 °C at 5 °C min^–1^. Oscillation was set to 0.50% strain and angular frequency of 2π rad/s.

Water contact angle measurements (AST Products VCA Optima) were recorded at an advancing and receding mode to calculate dynamic angles. Surface tension was calculated with a geometric-mean method.

Indentations and transfer printing were performed on the custom-built contact mechanics measuring system (CMMS) introduced in a previous work (Figure 1SI).^57^

Atomic force microscopy (AFM, NT-MDT Solver NEXT SPM) was used to measure surface topography and roughness using the semicontact mode.

An optical microscope (Carl Zeiss Axio Imager.A2m) was operated with varying magnifications (objectives: ×10–×50) and light sources (bright-field mode: Halogen 100-W, fluorescence mode: 200-W metal-halide lamp) to record surface indentations and thin-film transfers on the donor substrates and stamps.

A scanning electron microscope (SEM, Zeiss Auriga SEM/FIB) was operated with the InLens mode, 6 kV, and a working distance ranging from 4 mm to 8 mm. Images were recorded in the top view and at a 45° angle.

X-ray photoelectron spectrometer (XPS, Kratos AXIS Ultra DLD XPS) was equipped with a mono-Al X-ray source (1468.6 eV). The XPS spectra were collected over an ∼600 × 900 μm substrate area with multiple sweeps for the survey and regional scans to increase the signal-to-noise ratio. Unless specified, the electron collection angle θ in all XPS measurements was 90°. Casa XPS software was used for the data processing and analysis.

### Methods

#### Donor Substrate Fabrication and Regeneration

The fabrication process is shown in Figure 2a. A Si (100) wafer with a 500 nm thermal oxide layer was immersed in Nanostrip at 75° for 30 min, rinsed with isopropanol and DI water, and dried with N_2_ flow. A 2 μm layer of negative photoresist SU-8 was spin-coated onto the substrate and exposed under UV light with a photomask to form an array of 5 μm diameter circular features. After post bake (95 °C, 2 min) and development, the SU-8 micro disk array was used as a mask in reactive ion etching of the SiO_2_ layer. RIE was operated with a mixture of 42.5 sccm Ar, 2.5 sccm O_2_, and 5.0 sccm CHF_3_ gases at *P* = 140 mTorr and 100 W forward power. Twenty minute etching (5 min per step with 1 min pause intervals between each step to prevent heat accumulation) was applied to etch through the thermal oxide layer. After RIE, the substrate was immersed in Nanostrip for 1 h at 75 °C and cleaned with O_2_ plasma for 5 min at 100 W forward power to remove the remaining photoresist and other organic residues. The undercut structures were manufactured using KOH wet etching of the exposed Si(100) regions. As such, the cleaned substrate was first immersed in a 7:1 BOE solution at room temperature for 1 min to remove the native oxide layer. Subsequently, the substrate was immersed in 30% (wt%) KOH solution at 70 °C for 70 s and cleaned with DI water. After the wet etch, the substrate was sputtered with a 12 nm thick Au layer and functionalized with 1*H*,1*H*,2*H*,2*H-*perfluorodecanethiol solution in isopropanol (100 mmol/L, 12 h at 23 °C) to reduce its surface energy. The process yields a pattern of 3.7 μm SiO_2_ circular features on Si with 10 μm periodicity (Figure 2c). XPS was used to monitor the material transfer during the printing process and to analyze the composition of the donor substrate after cleaning and regeneration.

#### PUA_G_ Synthesis and Stamp Molding

The PUA monomer (**A**) was prepared from IPDI (0.01 mol), tin(II) 2-ethylhexanoate (0.0001 mol), 4,4′-methylenebis(2,6-di-*tert*-butylphenol) (0.0001 mol), PEG (0.05 mol), and hydroxypropyl acrylate (0.1 mol) by following a previously published protocol.^8^ Monomer (A) was then diluted with trimethylolpropane proxylate triacrylate (B, 40 v/v%) and HDODA (C 20 v/v%) to reduce viscosity and introduce reactive points for cross-linking. This mixture was added with the photoinitiators 1-hydroxycyclohexyl phenyl ketone (1 wt %) and 2-hydroxy-2-methyl-propiophenone (1 wt %).

To prepare flat SMP stamps, the mixture was degassed and poured into a flat-bottom mold with 2.2 mm spaces and covered with a glass substrate. The assembly was cured using a UV light lamp (UVP UVGL-15, 365 nm, 4 W) overnight and a UV cross-linkers system (SpectrolinkerTM XL-1500, 254 nm 6 × 15 W) for an additional 300 s. The sample was then cut into ∼5 mm × 5 mm flat stamps.

#### Thin-Film Deposition via Thermal Evaporation

A donor substrate was deposited with TCTA doped with FIrpic (2.8 + 1.2 Å/s, 60 nm, 30%), followed by aluminum (5–10 Å/s, 80 nm). Receiver substrate was deposited with MoO_3_ (1 Å/s, 1 nm) and TCTA (2.8 Å/s, 20 nm).

#### Compression Cycle and Transfer Printing Experiments

Donor/receiving substrates were placed in the middle of the stage, which has temperature control, position control, and force measurement. The elastomer stamp was attached to a microscopic glass slide with double-sided Kapton tape and mounted onto the substrate holder, with the stamp side facing the stage. The sample was compressed with 400 μm displacement against and detached from the donor/receiving substrates by the stage movement control, while the force data were continuously monitored. Pull-off work was calculated by integrating the area under the pull-off peak area in “force vs time” data while converting time to displacement using a constant stage movement rate of 5 μm/s. AFM measurements were taken for roughness and feature profiles. Optical microscopy and SEM were used for imaging the thin-film microstructures and calculating transfer yields using ImageJ. XPS data were taken to confirm the transfer and recycling steps with compositional analysis. The specific details of different compression and printing cycles are listed below:

##### Adhesion Measurement

Each stamp sample was processed with the following compression procedure, and the corresponding pull-off work was calculated by integration of calculated tension-versus-displacement curves using a pull-off rate of 5 μm/s.

Cold: Stamp was pressed against a clean donor substrate at room temperature for 10 min and then detached.

Hot: Stamp was pressed against a clean donor substrate at 80 °C for 10 min and then detached.

Thermomechanical cycle: Stamp was heated to 80 °C and compressed against a clean donor substrate for 10 min, cooled to room temperature for 1 h, and then detached from the donor substrate.

##### Microindentation and Shape Recovery

The stamp was processed through the thermomechanical cycle procedure with a clean donor substrate. Surface topography and roughness of the stamp were measured using AFM in a semicontact mode to confirm shape memory deformation. As evidence of shape recovery, a sequence of microscopic images of the stamp surface were recorded with an optical microscope while heating the stamp to 80 °C.

##### Transfer Printing with Thermomechanical Cycle

The stamp was processed through the thermomechanical cycle procedure with a donor substrate after thin-film deposition. The stamp with picked-up thin-film stacks was then brought into contact (<0.1 N/stamp) with the receiving substrate, heated to 80 °C for 25 min, and detached, leaving the films adhered to the receiving substrate. Optical microscopy with both bright-field and fluorescent modes was used to examine organic and metallic thin-film layers on the donor substrate, stamp, and receiver substrate after each step. The images were compiled and processed with ImageJ for yield calculation. SEM images were acquired as well to visually confirm the quality of the transferred features with a higher resolution and magnification. XPS was used to confirm successful transfer of the thin-film stacks.

##### Control Transfer Printing at Fixed Temperature

Stamps were processed through both the cold and hot compression procedures with donor substrates after the thin-film deposition. Both samples were imaged with an optical microscope in the bright-field mode. The images were compiled and processed with ImageJ for yield calculation.

## Supporting Information Available

Schematics and details of the custom-built contact mechanics measuring system (CMMS); high-resolution SEM images of the transferred features showing edge resolution (PDF)
