# Supplementary material for: Contact Printing of Multilayered Thin Films with Shape Memory Polymers
Source: ACS Nano. 2022 Mar 30;16(4):6134–44. doi: 10.1021/acsnano.1c11607 (PMC9047662; doi:10.1021/acsnano.1c11607)
Supplement: Supplementary file 1 — nn1c11607_si_001.pdf [file nn1c11607_si_001.pdf]

# Contact Printing of Multilayered Thin Films with Shape Memory Polymers

*Soyoun Kim, Nan Liu, Alexander A. Shestopalov**

*Department of Chemical Engineering, University of Rochester, Rochester, New York 14625, United States

## Supporting Information

**Contact Mechanics Measuring System (CMMS)**

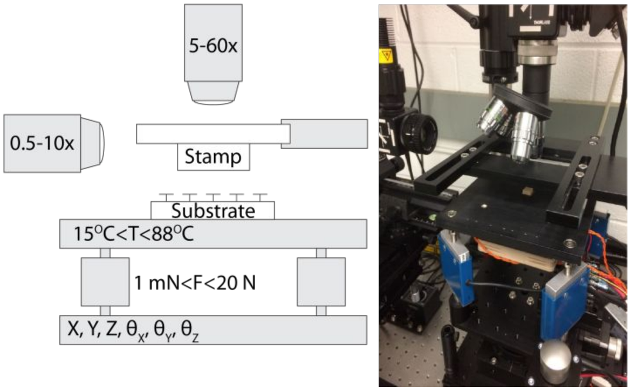


**Figure 1SI.** Schematics of contact mechanics measuring system (CMMS)

The system is equipped with a fixed glass substrate holder, XYZ-axis stepper/piezo motor (Thorlabs apt precision control BSC 203/303, range: 50mm/20μm, resolution: 0.5μm/20nm), a multi-channel amplifier with 4 load cells (Interface BSC8D-12, range: 5N per cell, resolution: 0.05%) for force monitoring, a thermoelectric heater (TE Technology TC-720, range: 15 ˚C – 88 ˚C, resolution: 0.01 ˚C), and custom-built lateral and vertical microscopes for profile and plan views.

The donor/receiver substrate was mounted with adhesive tape in the middle of the stage with temperature control, position control and force measurement. A clean microscope glass slide was mounted into the substrate holder, with the elastomeric stamp attached upside down, facing the substrate on the stage. Two orthogonally positioned microscope cameras were aligned with the substrate holder (profile view) and the polymer sample (plan view). The stamp was compressed against and detached from the glass slide by the stage movement control, while continuously monitoring the stage position, stamp-substrate contact area and force data. Pull-off work was

calculated by integration of calculated tension-versus-displacement curves using a pull-off rate of 5μm/s.
